# Pharmacokinetics, biodistribution and toxicology of novel cell-penetrating peptides

**DOI:** 10.1038/s41598-023-37280-0

**Published:** 2023-07-08

**Authors:** L. Reveret, M. Leclerc, F. Morin, V. Émond, F. Calon

**Affiliations:** 1 Faculty of Pharmacy, Université Laval, Quebec City, QC Canada; 2 Neurosciences Axis, CHU de Québec-Université Laval Research Center, 2705, Boulevard Laurier, Room T2-67, Quebec City, QC G1V 4G2 Canada

**Keywords:** Pharmacokinetics, Toxicology, Molecular medicine

## Abstract

Cell-penetrating peptides (CPPs) have been used in basic and preclinical research in the past 30 years to facilitate drug delivery into target cells. However, translation toward the clinic has not been successful so far. Here, we studied the pharmacokinetic (PK) and biodistribution profiles of Shuttle cell-penetrating peptides (S-CPP) in rodents, combined or not with an immunoglobulin G (IgG) cargo. We compared two enantiomers of S-CPP that contain both a protein transduction domain and an endosomal escape domain, with previously shown capacity for cytoplasmic delivery. The plasma concentration *versus* time curve of both radiolabelled S-CPPs required a two-compartment PK analytical model, which showed a fast distribution phase (t_1/2_α ranging from 1.25 to 3 min) followed by a slower elimination phase (t_1/2_β ranging from 5 to 15 h) after intravenous injection. Cargo IgG combined to S-CPPs displayed longer elimination half-life, of up to 25 h. The fast decrease in plasma concentration of S-CPPs was associated with an accumulation in target organs assessed at 1 and 5 h post-injection, particularly in the liver. In addition, in situ cerebral perfusion (ISCP) of L-S-CPP yielded a brain uptake coefficient of 7.2 ± 1.1 µl g^−1^ s^−1^, consistent with penetration across the blood–brain barrier (BBB), without damaging its integrity in vivo. No sign of peripheral toxicity was detected either by examining hematologic and biochemical blood parameters, or by measuring cytokine levels in plasma. In conclusion, S-CPPs are promising non-toxic transport vectors for improved tissue distribution of drug cargos in vivo.

## Introduction

Cell-penetrating peptides (CPPs) are small peptides (shorter than 30 amino acids), often cationic, with a capacity to penetrate the cell membrane^1^. The very first report of CPPs arose with the observation that the HIV-trans-activator of transcription (TAT) protein could reach the nucleus of cultured cells, resulting in target gene expression^2,3^. Subsequent studies showed that CPPs enhance cellular concentrations of cargos such as peptides, proteins, and DNA- or RNA-based medicines^4–8^. However, most studies with CPPs have been caried out in vitro. As with any drug, CPPs must also display acceptable pharmacokinetic (PK) and toxicity profiles to be translated toward in vivo and clinical uses.

Over 25 clinical trials involving CPPs are in progress including a few in Phase III^9^, the vast majority of them intended for application in oncology^10^. Yet, no CPP-based drugs have been approved by the US Food and Drug Administration (FDA). Although the efficacy of CPPs in bioassays have been the focus of many studies^11–14^, the knowledge on their PK properties and biodistribution in vivo is limited. The bulk of the work on CPPs has focused on a small number of entities, with TAT and penetratin representing 44% and 23% of the studies, respectively^15–19^. Biodistribution studies of different CPPs have shown preferential accumulation in highly perfused capillary-rich organs, such as the lungs, spleen, kidneys and liver^19^. Available PK studies suggest that CPPs undergo rapid liver and renal clearance, thereby reducing their plasma area under the curve and limiting subsequent target engagement.^19–23^. Regarding the safety profile of CPPs, most studies report that toxicity remains relatively low but may vary according to the types of CPP and cargos^24–27^.

As CPPs readily cross cellular membranes, the question arises whether they can also cross the blood–brain barrier (BBB), which protects the brain and prevents passage of the vast majority of macromolecules. However, studies available describing the ability of CPPs to reach the brain parenchyma are few. Stalmans et al., have investigated the BBB transport of 5 different CPPs using multiple-time regression analysis and showed that the majority display very high unidirectional influx rates^28^. CPPs are also used to improve brain-delivery formulations, such as targeted liposomes, in particular those aimed at transferrin receptor (TfR)-mediated delivery^29^. Encouraging results have been obtained with TfR-targeting liposomes coupled to CPPs that led to improved brain uptake^29–31^. In summary, a better understanding of PK and biodistribution properties of CPPs is needed for translation into clinical applications.

Here, we have studied a novel CPP comprising both a protein transduction domain (PTD) and an endosomal escape domain (EED) named Shuttle cell-penetrating peptide (S-CPP). This S-CPP has already been shown to undergo rapid, safe, and efficient cytosolic delivery of functional proteins into 20 mammalian cell types in vitro^32,33^. One of the major limitations in the development of CPPs is their entrapment with their cargo inside endosomes during intracellular trafficking, leading to lysosomal degradation^34^. EEDs can interact with the endosomal membrane to cause its degradation, its destabilization or pore formation, allowing leakage of endosomal content into the cytoplasm^35–37^. In addition, we have investigated the effect of the conformation of S-CPP by comparing its L and D enantiomers. D-amino acids are generally expected to protect from enzymatic activity and are thus more likely to increase peptide stability in vivo^38,39^. However, the CPPsite 2.0 database (https://webs.iiitd.edu.in/raghava/cppsite/index.html) shows that, out of 1850 sequences of referenced CPPs, less than 350 relate to D forms, suggesting that PK features of D-CPPs may be understudied*.* In summary, we have carried out PK and biodistribution studies of a S-CPP, alone or non-covalently combined to an immunoglobulin G (IgG) cargo, in its two enantiomeric forms, after systemic injection in animals. We have also evaluated toxicity from hematological, biochemical and inflammatory standpoints after repeated high-dose systemic injections in rodents. Finally, brain uptake of S-CPPs across the BBB was evaluated using in situ cerebral perfusion.

## Results

### Pharmacokinetic (PK) studies

#### Plasma PK profiles of the two S-CPP enantiomers

**Figure 1:**
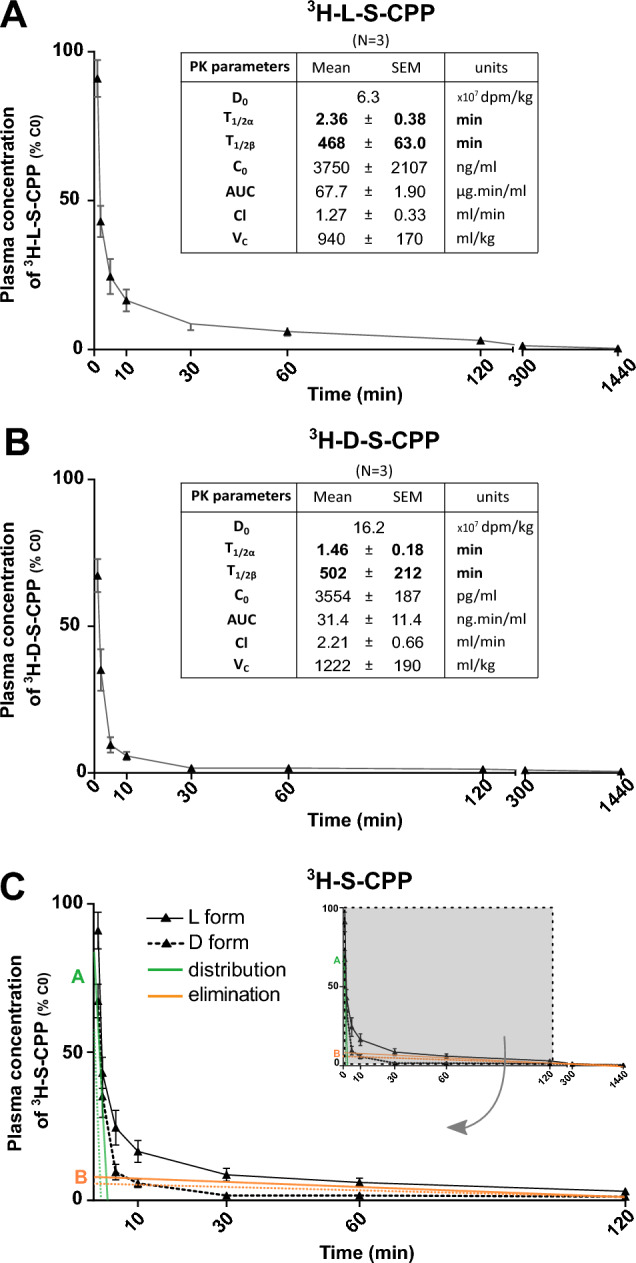
Pharmacokinetic profiles of enantiomers L- and D-S-CPP, according to a bicompartment model after a single intravenous injection in rats. Ten-month-old wild-type female Wistar rats were injected in the caudal vein at T_0_ and blood was collected at different time points until 24 h. Linear graphical representation of plasma concentrations of ^3^H-L-S-CPP (dose = 6.3 × 10^7^ dpm  kg^−1^, or 32.0 µg  kg^−1^) **(A)** and ^3^H-D-S-CPP (dose = 16.2 × 10^7^ dpm  kg^−1^, or 3.5 µg  kg^−1^) **(B).** Plasma concentrations are presented as the % of the respective calculated initial concentration (C_0_). Both sets of curves followed a bicompartment PK model with two phases: a rapid distribution phase (illustrated in green with intercept A as distribution coefficient) and a much longer elimination phase (illustrated in orange with intercept B as elimination coefficient) **(C)**. Data are presented as the mean ± SEM. *AUC* Area under the curve, *C*_*0*_ initial estimated concentration, *Cl* clearance*, D*_*0*_ initial dose, *dpm* disintegration per minute, *S-CPP* Shuttle cell-penetrating peptides, *T*_*1/2α/β*_ half-life of distribution (α) and elimination (β), *Vd* estimated area of the compound’s distribution.

PK analyses were based on plasma data following a single intravenous (i.v.) injection in the caudal vein of rats (Table 1). Plasma concentrations *versus* time curves and PK parameters of the S-CPPs L-S-CPP and D-S-CPP are plotted in Fig. 1. In the case of i.v. bolus administration, C_max_ is equal to concentration at time zero (C_0_), extrapolated from the curve at the y-intercept. As expected, the PK concentration–time pattern of the S-CPPs required a two-compartment model, separating distribution and elimination phases. Plasma counting of ^3^H-L-S-CPP or ^3^H-D-S-CPP showed a fast distribution phase (T_1/2_α ranging between 1.3 and 3 min) followed by a slower elimination phase (T_1/2_β ranging between 4 and 15 h) after i.v. injection. Central volumes of distribution (V_C_ > 900 mL/kg) were many times more elevated than whole plasma volumes in the rat. These results are consistent with a quick distribution of S-CPPs in organs when injected alone. Finally, although doses, AUC and C_0_ were different due to the necessity to inject sufficient amounts of radioactivity to all animals, both S-CPPs had similar T_1/2_, T_max_, Cl and V_C_, suggesting no major difference in elimination and distribution.

**Table 1:** Experimental design and sample collection.

	Animal	Substance	N/group	Doses	Time points
Pharmacokinetic profiles	Rats	^3^H-L-S-CPP	3	6.3 × 10^7^ dpm kg^−1^ (D_o_) (32.0 ug kg^−1^)	0.016, 0.033, 0.083, 0.166, 0.5, 1, 2, 5, and 24 h after administration
		^3^H-D-S-CPP	3	16.2 × 10^7^ dpm kg^−1^ (D_o_) (3.5 ug kg^−1^)	
		^3^H-IgG	3	12.8 × 10^6^ dpm kg^−1^ (D_o_) (19.1 ug kg^−1^) ± L-S-CPP 1.4 mg kg^−1^ (250 µM)	0.016, 0.033, 0.083, 0.166, 0.5, 1, 2, 5, 24 and 48 h after administration
		^3^H-IgG + L-S-CPP	3		
Biodistribution	Mice	^3^H-L-S-CPP	2–4 per group	10.4–15.9 ug kg^−1^	1 and 5 h after administration
		^3^H-D-S-CPP			
		^3^H-IgG_antiNUP_	6	82.5 ug kg^−1^ ± L/D-S-CPP or Scramble ≈ 3.6 mg kg^−1^	1 h after administration
		^3^H-IgG_antiNUP_ + L-S-CPP	11–15 per group		
		^3^H-IgG_antiNUP_ + D-S-CPP			
		^3^H-IgG_antiNUP_ + Scramble			
Toxicology	Mice	L-S-CPP	4–5 per group	3.6 mg kg^−1^	120 h
		D-S-CPP			
		PBS			
BBB Transport	Mice	^3^H-L-S-CPP	15	0.2–0.8 µCi ml^−1^ Corresponding to 0.2–0.8 µg (≈7.0-27 µg kg^−1^)	60 s

*BBB* blood brain barrier, *D*_*0*_ initial dose (in syringe), *S-CPP* Shuttle-cell penetrating peptides, *h* hour, *IgG* immunoglobulin G, *IgG*_*antiNUP*_ anti-Nuclear Pore Complex Proteins Antibody, *PBS* Phosphate-buffered saline.

#### PK parameters of IgG are not changed by combination with S-CPP.

**Figure 2:**
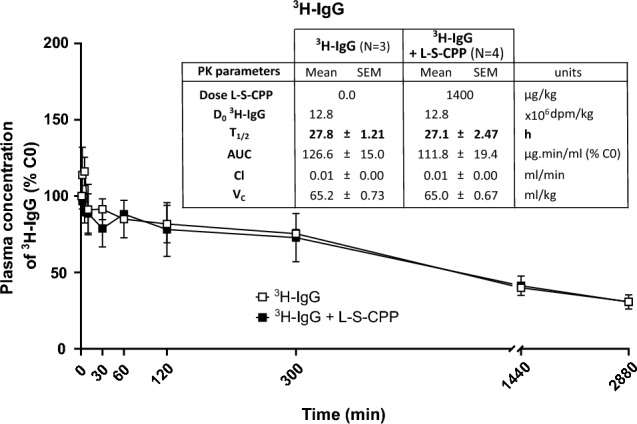
Pharmacokinetic profile of ^3^H-IgG combined to L-S-CPP, according to a linear model after a single intravenous injection in rats. Ten-month-old wild-type female Wistar rats were injected in the caudal vein at T_0_ and blood was collected at different time points until 48 h. Linear graphical representation of plasma concentrations of ^3^H-IgG (at 1.3 × 10^7^ dpm  kg^−1^ = 19.9 µg.kg^- 1^, or with L-S-CPP (1.4 mg  kg^−1^). Plasma concentrations are represented as the % of the respective calculated initial concentration (C_0_). Both sets of curves followed a linear PK model. PK parameters are shown in the inserted Tables. Data are presented as mean ± SEM. Statistical analysis: Student’s t-test between ^3^H-IgG alone compared to the combination with L-S-CPP at equivalent doses for calculated PK parameters. *AUC* Area under the curve, *C*_*0*_ initial estimated concentration, *Cl* clearance, *D*_*i*_ initial theorical dose, *dpm* disintegration per minute, *Dose*_*extrapolated*_ graphically estimated dose, *S-CPP* Shuttle cell-penetrating peptides, *IgG* immunoglobulin G, *T*_*1/2*_ half-life, *V*_*D*_ estimated area of the compound’s distribution.

The PK parameters of ^3^H-IgG administered alone or combined with L-S-CPP are shown in Fig. 2. Plasma concentration–time curves were linear and required single-compartment models to estimate the PK parameters. The calculated elimination half-life was over 25 h, consistent with a long blood residence time. The central volume of distribution was closer to plasma volumes in the rat (V_C_ ≈ 65 mL/kg), consistent with retention into the blood compartment. Combination with L-S-CPP did not change the PK parameters measured for each IgG. As expected, the C_0_ of L-S-CPP-IgG was higher than IgG due to the difference in dose.

### Biodistribution studies

#### S-CPPs are quickly distributed into organs

**Figure 3:**
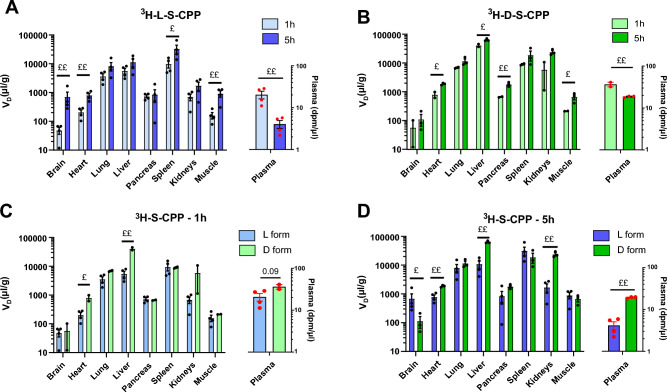
Apparent volume of distribution (μl  g^−1^) of tritiated enantiomers L- and D-S-CPP, 1 and 5 h after intravenous injection. Ten-week-old male CD-1 mice were injected in the caudal vein and sacrificed by intracardiac perfusion 1 or 5 h post injection of ^3^H-L-S-CPP = 10.4 µg  kg^−1^ **(A)** or ^3^H-D-S-CPP = 15.9 µg  kg^−1^** (B)** Tissues were homogenized and comparisons between the two forms of S-CPP are illustrated **(C**,**D)**. The apparent volume of distribution (μl  g^−1^) in each organ was calculated by dividing radioactive counts (dpm  g^−1^) of each tissue by plasma counts (dpm  µl^−1^) at the same time point. Data are represented on a logarithmic scale with a mean of N = 2–4 ± SEM. Statistical analyses were performed on values ​​after logarithmic transformation with an unpaired Student t-test (^£^p < 0.05; ^££^p < 0.01; ^£££^p < 0.001). *dpm* disintegration per minute, *S-CPP* Shuttle cell-penetrating peptides, *V*_*D*_ estimated area of the compound’s distribution.

We then investigated the volumes of distribution of S-CPP enantiomers ^3^H-L-S-CPP and ^3^H-D-S-CPP 1 h and 5 h after an i.v. injection in the tail vein of mice. The apparent volume of distribution (V_D_, expressed in µl g^−1^) was determined by dividing the radioactivity in each harvested organ, expressed in dpm  g^−1^, by the radioactivity in the plasma expressed in dpm µl^−1^ (Fig. 3). Radioactivity from the tritiated S-CPP was detected in all organs 1 h and 5 h after i.v. injection in the tail vein (Fig. 3A,B). The lung, liver, and spleen had the highest levels of S-CPPs for both enantiomers (Fig. 3A,B). This was expected, as CPPs were shown to accumulate in highly vascularized organs^19^. The plasma concentrations of ^3^H-L-S-CPP and ^3^H-D-S-CPP decreased from 1 to 5 h after i.v. injection. However, the V_D_ was higher at 5 h than at 1 h in several organs such as the brain, heart, muscle, and spleen (Fig. 3), suggesting a slower clearance in these organs than in the blood. The comparison between L and D enantiomers of S-CPP is shown in Fig. 3C,D. Both enantiomers followed the same pattern at 1 h post i.v. injection, with a predictable accumulation in the liver and spleen. One hour after administration, we observed that the V_D_ of the D form was higher in the heart and liver than the L enantiomer (Fig. 3C). After five hours, higher relative contents of the D form were also found in the plasma and the kidneys. This contrasts with the brain where a higher V_D_ was detected for the L enantiomer. TCA precipitation of tritiated S-CPPs from the liver, the organ that showed the highest accumulation, indicates that 83.1% (± SD 16.4%, n = 3) of the radioactivity measured was still associated with S-CPPs after 5 h.

#### Combination with S-CPP increases the uptake of ^3^H-IgG in the liver

**Figure 4:**
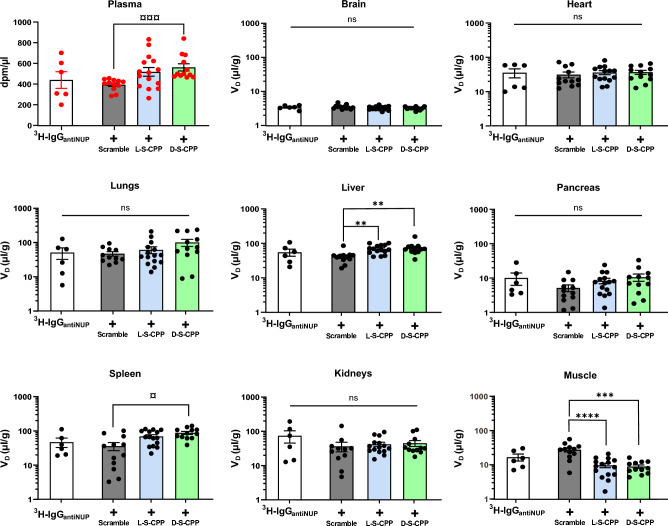
Apparent volume of distribution (μl g^−1^) of tritiated IgG_antiNUP_ 1 h after co-injection with either forms of S-CPP or a scrambled peptide. Ten-week-old male CD-1 mice were injected in the caudal vein and sacrificed by intracardiac perfusion at 1 h post injection, thereby removing blood from the brain. IgG_antiNUP_ targets nuclear pore proteins. The ^3^H-IgG_antiNUP_ dose was 82.5 µg  kg^−1^ ± L/D-S-CPP or combined with a control peptide ("Scramble" or SCR) at 3.6 mg  kg^−1^. The apparent volume of distribution (μl g^−1^) in each organ was calculated by dividing radioactive counts (dpm g^−1^) of each tissue by plasma counts (dpm µl^−1^). Data are represented on a logarithmic scale with the mean of N = 6–12 ± SEM. Statistical analyses were performed on values ​​after logarithmic transformation by a One-Way ANOVA parametric test followed by a Tukey post-hoc test (**p < 0.01; ***p < 0.001; ****p < 0.0001), a Welch ANOVA parametric test followed by a Dunnett’s post-hoc test ^(¤^p < 0.05; ^¤¤¤^p < 0.001). *ns* not significant, *dpm* disintegration per minute, *S-CPP* Shuttle cell-penetrating peptides, *IgG* immunoglobulin G, *V*_*D*_ estimated area of the compound’s distribution.

In order to elucidate whether combination with S-CPP alters the biodistribution of a large cargo, we compared the V_D_ in organs 1 h after systemic administration of ^3^H-IgG_,_ combined or not to unlabeled L-S-CPP, D-S-CPP, or a scrambled peptide (Fig. 4). Combining the ^3^H-IgG with D-S-CPP led to significantly higher plasma concentrations at 1 h post injection, when compared to ^3^H-IgG combined with the scrambled control peptide. This effect was not significant for L-S-CPP. The combination of ^3^H-IgG_antiNUP_ to S-CPPs increased its V_D_ in the liver but decreased its V_D_ in the muscle (Fig. 4). A higher relative distribution was also seen in the spleen but only for D-S-CPP (Fig. 4). No other significant difference was measured in other organs (Fig. 4). These data suggest that S-CPPs induced a preferential distribution of IgG in the liver and possibly spleen, but had the opposite effect in the muscle.

### Transport of L-S-CPP across the blood–brain barrier (BBB)

**Figure 5:**
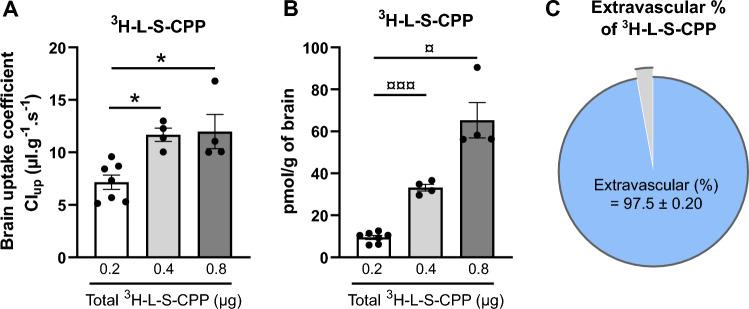
L-S-CPP crossed the blood–brain barrier, as assessed with in situ cerebral perfusion (ISCP). (**A**) The brain uptake coefficient (Clup; µl s^−1^ g^−1^) of ^3^H-L-S-CPP was calculated as V_brain_/T, where T is the time perfusion (60 s). Injected concentrations were: 0.08, 0.16 and 0.32 µg/ml, corresponding to doses per mice of 0.2, 0.4 and 0.8 µg. The rate of entry of ^3^H-L-S-CP in the brain did not decrease with higher concentrations, consistent with the absence of a saturable transport mechanism. (**B**) The pmol/g of ^3^H-L-S-CPP in the brain were estimated from the dpm/g (L-S-CPP) and the specific activity and showed a linear increase (r^2^ = 0.88; p < 0.0001), in accordance with free diffusion across the BBB. (**C**) Comparison of the proportion of ^3^H-L-S-CPP found in the vascular or extravascular fractions of the brain, showing a fast distribution into the brain parenchyma. Data are represented with the mean of N = 4–7 ± SEM. Statistical analyses were performed by a One-Way ANOVA parametric test followed by a Tukey post-hoc test (*p < 0.01) or a Welch ANOVA parametric test followed by a Dunnett’s post-hoc test (^¤^p < 0.05; ^¤¤¤^p < 0.001). *Clup* brain uptake coefficient, *S-CPP* Shuttle cell-penetrating peptides.

The biodistribution studies revealed a V_brain_ of 45.6 ± 13.1 µl.g^−1^ and 715.1 ± 335.0 µl  g^−1^, at 1 h and 5 h respectively, for ^3^H-L-S-CPP (Fig. 3), consistent with a relative cerebral accumulation of ^3^H-L-S-CPP. To better characterize the passage of this CPP through the BBB, we performed ISCP to infuse directly ^3^H-L-S-CPP into the carotid at a dose of 0.2 μg corresponding to a concentration of 0.08 μg/ml. The observed brain coefficient uptake (Clup) of ^3^H-L-S-CPP (7.2 ± 1.8 µl  g^−1^ s^−1^) indicates a relatively high capacity of S-CPPs to cross the BBB (Fig. 5A). To gain further insights on the mechanism of transport, we used increasing concentrations of ^3^H-L-S-CPP (0.08, 0.16 and 0.32 μg/ml). The absence of saturation in the rate of transport across the BBB (Fig. 5A) suggests that L-S-CPP transport across the BBB probably does not involve receptor-mediated endocytosis but mostly simple diffusion. The total pmol/g of ^3^H-L-S-CPP collected in the brain increased linearly with rising concentrations of ^3^H-L-S-CPP perfused (r^2^ = 0.88, p < 0.0001; Fig. 5B), which also indicates that L-S-CPPs do not use a saturable mechanism. Moreover, the extravascular % of ^3^H-L-S-CPP was 97.5 ± 0.3%, which is consistent with a fast distribution outside of the vascular compartment (Fig. 5C). Finally, the Vvasc measured in each mouse using ^14^C-sucrose remained in the normal range (lower than 20 µl  g^−1^), confirming that the CPPs did not alter the integrity of the BBB.

### Toxicology studies

To assess their toxicity, we have injected high doses of L-S-CPP and D-S-CPP (each at 3.6 mg kg^−1^) in the tail vein of mice, BID for 5 days. Mice did not lose weight during this time period. A blood sample was collected at the end of the experiment for each animal and hematological and biochemical analyses were performed to evaluate toxicity. The data revealed no abnormalities, as observable differences between mice remained within normal ranges (Tables 2 and 3).

**Table 2:** Blood hematology parameters after chronic high dose of S-CPP.

			PBS (n = 5)	L-S-CPP (n = 7)	D-S-CPP (n = 8)
Hematology		Units	Mean ± SEM	Mean ± SEM	Mean ± SEM
White Blood Cells	WBC	10^3^/mm^3^	7.1 ± 2.2	6.5 ± 0.9	4.4 ± 0.4
Lymphocytes	LYM	10^3^/mm^3^	3.0 ± 1.1	3.1 ± 0.4	1.9 ± 0.1
Monocytes	MON	10^3^/mm^3^	0.4 ± 0.4	0.3 ± 0.2	0.1 ± 0.1
Granulocytes	GRA	10^3^/mm^3^	3.6 ± 1.1	2.9 ± 0.4	2.2 ± 0.2
Eosinophils	EOS	10^3^/mm^3^	0.8 ± 0.5	0.5 ± 0.2	0.1 ± 0.0
% Lymphocytes	LYM	%	43.9 ± 3.7	48.7 ± 1.5	43.8 ± 2.6
% Monocytes	MON	%	7.4 ± 1.2	9.2 ± 0.9	9.7 ± 0.7
% Granulocytes	GRA	%	48.7 ± 3.0	42.1 ± 1.8	46.5 ± 2.3
% Eosinophils	EOS	%	7.7 ± 3.7	5.7 ± 2.0	2.2 ± 0.3
Red Blood Cells	RBC	10^6^/mm^3^	8.8 ± 0.3	7.8 ± 0.3	7.3 ± 0.5
Hemoglobin	HGB	g/dl	13.8 ± 0.5	14.0 ± 0.5	12.9 ± 0.8
% Hematocrit	HCT	%	40.6 ± 1.4	38.3 ± 1.1	35.5 ± 2.6
Mean Corpuscular Volume	MCV	µm^3^	46.0 ± 0.5	49.4 ± 0.6	49.0 ± 0.6
Mean Cell Hemoglobin	MCH	pg	15.6 ± 0.2	17.9 ± 0.4	17.9 ± 0.5
Mean Cell Hemoglobin Concentration	MCHC	g/dl	33.9 ± 0.1	36.5 ± 0.8	36.6 ± 0.8
Red Cell Distribution Width	RDW	%	14.8 ± 0.3	13.9 ± 0.1	14.1 ± 0.2
Platelets	PLT	10^3^/mm^3^	884.2 ± 132.5	775.1 ± 77.7	843.3 ± 108.3
Mean Platelet Volume	MPV	µm^3^	5.3 ± 0.1	5.7 ± 0.1	5.7 ± 0.1

Mice were injected i.v. twice a day for 5 days (total of 10 injections) and sacrificed by intracardiac perfusion. Injected dose was 3.6 mg  kg^−1^. Data are presented as the mean ± SEM. The statistical analysis did not reveal any significant difference.

**Table 3:** Blood biochemical parameters and concentrations of pro- and anti-inflammatory cytokines after chronic high dose of S-CPP.

		PBS (n = 5)	L-S-CPP (n = 7)	D-S-CPP (n = 8)
Biochemistry	units	Mean ± SEM	Mean ± SEM	Mean ± SEM
Glucose	mmol/L	16.5 ± 0.4	14.8 ± 1.1	15.4 ± 1.1
Blood Urea Nitrogen	mmol/L	7.8 ± 0.0	6.3 ± 0.0	6.9 ± 0.0
Creatinine	mmol/L	18.0 ± 0.9	18.0 ± 0.4	18.0 ± 0.4
Phosphate	mmol/L	2.8 ± 0.2	3.0 ± 0.2	2.8 ± 0.1
Calcium	mmol/L	2.3 ± 0.1	2.6 ± 0.2	2.5 ± 0.0^££££^
Total protein	g/L	46.8 ± 1.2	52.4 ± 0.1	50.6 ± 1.4
Albumin	g/L	20.0 ± 0.6	23.4 ± 0.7^$$^	21.9 ± 0.7
Alanine Transaminase	U/L	35.4 ± 3.8	82.0 ± 11.5^$$^	99.9 ± 17.2^$$$^
Alkaline Phosphatase	U/L	42.8 ± 4.5	54.9 ± 5.3	66.4 ± 6.8^£^
γ-Glutamyl Transferase	10^6^/mm^3^	10.0 ± 0.0	10.0 ± 0.0	10.0 ± 0.0
Total Bilirubin	µmol/L	1.0 ± 0.0	2.3 ± 0.5	1.4 ± 0.4
Cholesterol	mmol/L	4.1 ± 0.1	4.85 ± 0.3	4.5 ± 0.1^££^

Cytokines (pg/mL)		Mean	Mean	Mean
Interleukin 1β	IL-1β	OOR <	OOR < or 3.3	OOR <
Interleukin 2	IL-2	OOR < or 0.4	OOR < or 0.8	OOR < or 0.9
Interleukin 4	IL-4	OOR <	OOR <	OOR <
Interleukin 5	IL-5	OOR < or 2.7	OOR < or 6.0	OOR < or 5.0
Interleukin 10	IL-10	OOR < or 12.5	OOR < or 23.3	20.3
Granulocyte–macrophage colony-stimulating factor	GM-CSF	OOR <	OOR <	OOR <
Interferon γ	INF-γ	3.0	2.9	4.0
Tumor necrosis factor α	TNFα	44.7	44.7	26.1

Mice were injected i.v. twice a day for 5 days (total of 10 injections) and sacrificed by an intracardiac perfusion. Normal levels of γGT (γ Glutamyl Transferase, U/l) and TB (total bilirubin, µmol/l) do not exceed 10, whereas creatinine should not go over 18 µmol/l. Injected dose was 3.6 mg  kg^−1^. Biochemistry data are presented as the mean ± SEM, and cytokines as the mean of duplicates (PBS) or triplicate (L/D-S-CPP) in pg/ml when it was possible because most data were out of range (OOR) and extrapolated by the software. Statistical analysis: Unpaired t-test (^£^p < 0.05, ^££^p < 0.01; ^££££^p < 0.0001) or Mann–Whitney (^$$^p < 0.01; ^$$$^p < 0.001), PBS *versus* S-CPP injected. The differences measured remained within normal ranges for a mouse. *Pro-inflammatory cytokines : TNF-α, IFN-ɣ, GM-CSF, IL-1ß, IL-2 and anti-inflammatory cytokines : IL-5, IL-4, IL-10.* Note: Overall values are very low. Certain points stand out for TNFα but the vast majority of values are near the detection limit.

Serum cytokine quantification using multiplex ELISA was also evaluated. A large panel of cytokines were measured in triplicates in plasma samples: TNF-α, IFN-ɣ, GM-CSF, IL-1ß, IL-2, IL-5, IL-4 and IL-10. Most pro-inflammatory cytokines or anti-inflammatory cytokines were below detection threshold. Therefore, despite the high doses used (in the mg/kg range), S-CPPs did not induce systemic inflammation or immunogenicity (Table 3).

## Discussion

Many potential therapeutic targets are located inside of cells; however, these targets often remain out of reach because therapeutic molecules must cross several physiological barriers, including ultimately the cytoplasmic membrane. The central nervous system (CNS) is particularly well protected by the blood–brain barrier (BBB), which blocks most biopharmaceuticals. For some decades, CPPs have been proposed as vehicles for intracellular delivery and solutions for this pharmaceutical challenge. The emergence of CPPs in the clinic has been impeded by the lack of preclinical PK-BD data. The aim of the present study was to investigate PK and BD parameters of CPPs alone or combined in vivo as well as their toxicity and brain uptake.

After intravenous administration, we observed that both S-CPPs displayed a fast distribution phase of less than 3 min. This is consistent with a quick distribution in organs due to the small size and relative lipophilicity of the CPPs^19^. Although a significant portion was rapidly removed from the blood, S-CPPs then displayed a longer and slower elimination phase (T_1/2_ over 450 min), whichwas uncovered because of the use of a two-compartment PK model. True in vivo PK studies of CPPs in the literature are scarce. For example, one study using a panel of ten CPPs report half-lives ranging from 72 (penetratin) to 528 (TAT) min to over 72 h by measuring their stability in vitro at 37 °C in human serum^19^, which does not take into account tissue distribution and metabolism, as well as excretion. On the other hand, Lee et al. reported T_1/2_ values of 0.87 and 107 min for the distribution and elimination phases, respectively, after i.v. injection of TAT-biotin in rats^19,40,41^. The present results are in line with these studies and suggest that S-CPPs alone may display a sufficient residence time to exert a pharmacological effect in target tissue.

Very few studies have compared CPP enantiomers in vivo^19,22,42,43^. The rationale for their comparison here was that stereoselective interactions with liver enzymes or the cell membrane might differ between L-S-CPP and D-S-CPP^44,45^. For example, it has been postulated that D-form CPPs may be more resistant to degradation by enzymes compared to L-forms, thereby increasing their stability in vivo^38,39^. Here, the D-form exhibited slightly lower T_1/2_ and AUC, as well as higher clearance and V_c_, compared to the L-form, although these differences did not reach statistical significance. Thus, our results do not suggest obvious advantages of using either enantiomer to improve PK parameters, although biodistribution was affected (see below).

As S-CPPs are designed to deliver protein-based cargo, PK parameters of intravenously injected immunoglobulin G (IgG), in the presence or not of S-CPPs, were investigated. As expected, the calculation of PK parameters of IgG required a single-compartment model because of the slow distribution and stability of IgG in the blood in vivo. Comparison of IgG alone or combined to L-S-CPP did not reveal significant differences in PK parameters such as AUC, clearance or V_D_. This suggests that the combination to CPPs did not modify cargo functionality. Thus, the PK data obtained in this study is consistent with what is expected for IgG, with a half-life of days to weeks in the mouse^46,47^. Interestingly, with regards to biodistribution, D-S-CPP increased the plasma concentration of IgG 1 h post-injection in mouse when compared to the scramble peptide. This indicates that IgG/S-CPP formulations may present an advantageous circulating time, increasing the propensity to reach their intracellular target after systemic administration.

The biodistribution studies provided a comparative view of the concentration of S-CPP reaching different organs at two different time points selected during the elimination phase. The apparent volume of distribution increased between 1 and 5 h post-injection in many organs, suggesting a relative accumulation over time. While this was true for both enantiomers in the heart and muscle, L-S-CPP displayed a preferential relative distribution in the brain and spleen, whereas for D-S-CPP it was the liver and pancreas. The comparison of the two enantiomers at 5 h shows that the D form was relatively more concentrated in the plasma, heart, liver and kidneys than the L form. This is consistent with previous work suggesting a greater stability of D-CPPs in vivo^39^ and suggests that the enzymatic resistance of D amino acids is noticeable only after several hours. A notable exception was the brain, in which the L-S-CPP exhibited a higher apparent volume of distribution than the D-form at 5 h post-injection. This tells us that, despite a reduction in plasma levels of both L-S-CPP and D-S-CPP between 1 and 5 h, concentrations in key organs remained at appreciable levels at both time points. For example, based on values in dpm/g and specific activity in dpm/µg, concentrations of S-CPP in the liver ranged between 2 and 10 nM up to 5 h post injection. The integrity of the S-CPPs (> 80%) in the liver was confirmed using TCA precipitation. Even in the brain, the levels of L- and D-S-CPP reached the low nanomolar zone (0.17 and 0.02 nM, respectively). Overall, the data presented indicates that S-CPPs can reach therapeutically relevant concentrations in target organs after systemic administration.

In future clinical applications, S-CPPs are unlikely to be utilized alone and their efficacy will ultimately be determined by the ability of their cargo to reach target organs^22,32^. The biodistribution of IgG_antiNUP_ was investigated with or without combined S-CPPs or a scrambled peptide. The combination with D-S-CPP increased plasma concentrations of the IgG at 1 h post-injection, suggesting a slower elimination not apparent in the previous PK experiment. The most striking observation was that both S-CPPs induced a preferential distribution of IgG in the liver but had the opposite effect in the muscle. A higher accumulation of IgG combined with D-S-CPP was also observed in the spleen compared to the scramble peptide. There is published evidence that D-penetratin improved nasal absorption of interferon beta (IFN-β) better than the L form after intranasal administration^48^. Here, concentrations of cargo IgG were estimated to be between 0.3 and 0.5 nM in the liver and spleen 1 h after administration. It should be reminded, however, that combining a CPP to a cargo smaller than an IgG may have had more impact on its distribution. Nevertheless, such an increase in accumulation of S-CPP/IgG complexes in the liver and spleen, while avoiding the muscle, may be therapeutically valuable for certain indications.

Considering their smaller size (up to 30 amino acids in length), cationic and/or amphipathic CPPs have a greater potential to penetrate the BBB than other transport systems^49^. Previous studies have suggested that most CPPs do not have access to the CNS^14^, in part because of their low AUC^41^. Here, we observed that L-S-CPP accumulated preferentially in the brain at 1 and 5 h post-injection. Using in situ cerebral perfusion (ISCP) to directly assess its capacity to cross the BBB, we calculated a Clup of approximately 7 µl  g^−1^  s^−1^. For the sake of comparison, a control IgG, an IgG binding the transferrin receptor (receptor-mediated endocytosis), glucose (facilitated transport) and diazepam (passive diffusion) display Clup of ≈0.005, ≈0.03, ≈1 and ≈40 µl  g^−1^  s^−1^, respectively^47,50–52^. Importantly, the infusion of 0.8 μg/mouse (80 nM) of CPP into the carotid did not impair the integrity of the BBB. The results from ISCP experiments showed that the rate of transport of L-S-CPP is relatively high and maintained or even increased with escalating concentrations. This indicates that the transport of CPPs across the BBB is not saturable and could be explained at least in part by free diffusion. The data is also consistent with a saturable efflux of L-S-CPP back to the blood. However, as most CPPs penetrate cells through more than one mechanism^53,54^ we cannot exclude that S-CPP use specific transport mechanisms in the presence of a cargo or under other experimental conditions. This is consistent with the hypothesis that S-CPPs activate translocation and endocytosis in a dose-dependent manner^32^. In addition, S-CPPs show affinity for heparan sulfate proteoglycans^32^, a type of membrane-bound entity present in endothelial cells of the BBB^55^. Further studies are needed to determine the exact mechanism of transport into the brain.

Owing to their physicochemical properties, CPPs can be internalized by almost any type of cell. However, few studies have addressed the toxic and immunological responses to CPPs in vivo. Here, hematologic and cytokine endpoints did not reveal any difference between treatment and control groups. Regarding biochemistry parameters, levels of alanine transaminase for both treated groups were higher than controls. L-S-CPP-injected mice also exhibited higher levels of albumin compared to phosphate-buffered saline (PBS)-injected mice. Yet, all of these levels remained in the normal range for a mouse^56^. In addition, no animal exhibited obvious changes in physical appearance, activity level, or body weight. Although no signs of toxicity were observed in the present study, previous studies have suggested that CPPs might act as double edge swords mediating a wide variety of unpredictable biological effects^38,57–59^. For this reason, we cannot fully rule out potential toxicity despite the high doses used in this study.

In summary, S-CPPs PK, biodistribution and toxicity data gathered here argue in favor of their potential use in vivo, particularly when combined to cargos with long residence time in circulation. Therapeutically relevant distributions of S-CPPs were reached in multiple organs, such as the liver, the spleen and the kidney, but also the CNS for uncombined L-S-CPP. The present results therefore suggest that the capacity of S-CPPs to improve target engagement of biopharmaceuticals after systemic administration should be further investigated.

## Methods

### Materials and radioactive labelling

Peptides used in this study were synthesized by GL Biochem (Shanghai, China), as described^32,33,60,61^. They performed the purification by reversed-phase high performance liquid chromatography and confirmed peptide identity by mass spectrometry (Agilent-6125B). Purity reached 95%. L-S-CPP and D-S-CPP are the two enantiomers of S-CPP with the following properties:

**Table:** 

Molecular weight	Number of amino acids	Ratio of hydrophilic amino acids	Average hydrophilicity	Isoelectric point	Net charge at pH 7
3352 g mol^−1^	30	40%	0.07	12.48	8 +

Peptide sequences are proprietary to Feldan Therapeutics^60^ and include a protein transduction domain (PTD) and an endosomal escape domain (EED) (Patent No.; International Publication Number, WO2022204806A1, WO2022082315A8, WO2017175072A1, WO2022077121A1). Tested cargos were immunoglobulins G (IgG, molecular weight ≈ 150 000 g mol^−1^). We used a mouse monoclonal IgG recognizing intracellular anti-nuclear pore complex proteins (IgG_antiNUP_) purchased from BioLegend, as well as a rat IgG possessing no mammalian reactivity (IgG_control_) purchased from Bio-X-Cell. CPPs and cargoes were co-incubated for at least 30 min in PBS to achieve non-covalent combination.

L-S-CPP and IgGs were radiolabeled with N**-**Succinimidyl propionate-2,3-[^3^H] (^3^H-NSP, Moravek). Briefly, ^3^H-NSP was separated in 1-mCi aliquots, evaporated under nitrogen, resuspended in bicarbonate buffer (NaHCO_3_ 0.1 M pH 8.3) containing L-S-CPP (44 nmoles), IgG_antiNUP_ (8 nmoles) or IgG_control_ (8 nmoles), and incubated with shaking for 5 h at room temperature. Radiolabeled molecules were purified by dialysis with Micro Float-A-Lyzer (L-S-CPP) or Vivaspin centrifugal devices (IgGs) and kept at − 20 °C in 50% glycerol. The specific activity of ^3^H-L-S-CPP, ^3^H-IgG_antiNUP_ and ^3^H-IgG_control_ were 2.48, 0.31 and 0.29 µCi µg^−1^, respectively. The purity of radiolabeled S-CPP and IgGs was confirmed by 20% trichloroacetic acid (TCA) precipitation where over 99% of tritium counts were consistently observed in the precipitated fraction. Tritiation of D-S-CPP (0.7 mCi ml^−1^) was performed by RC TRITEC AG (Teufen, Switzerland) and the specific activity was 20.7 µCi µg^−1^. ^14^C-sucrose (1.5 µCi µg^−1^) was purchased from Moravek Biochemicals (Brea, CA, USA).

Tissue samples underwent digestion in SOLVABLE solubilizer and counting in Ultima Gold scintillation cocktail both purchased from PerkinElmer (Waltham, MA, USA). All isotopes were counted in a Hidex 300 SL Liquid Scintillation Counter or Wallac 1409 Liquid Scintillation Counter.

### Animals

All rodents were purchased from Charles River. Animals were allowed access to food and water ad libitum. Mice and rats were fed with chow diet (2018 Teklad global 18% protein). All experiments were performed in accordance with the Canadian Council on Animal Care and were approved by the Institutional Animal Care Committee of Université Laval. Study details are in accordance with ARRIVE guidelines.

### Pharmacokinetic (PK) studies

Wistar rats were used instead of mice to collect a sufficient volume of blood at different timepoints in the same animal, thus reducing inter-animal variability and sparing animals, in agreement with suggestions from our Animal Ethics Committee. Rats (10-month-old females) were anesthetized with isoflurane and injected in the caudal vein at T_0_. Blood samples were collected at the jugular vein at different time points until 24 or 48 h (Table 1). Blood samples were centrifuged, and plasma was counted for both total and TCA-precipitable radioactivity per volume. Rats were sacrificed using a carbon dioxide (CO_2_) chamber. Injected doses were: ^3^H-L-S-CPP = 6.3 × 10^7^ dpm  kg^−1^ (32.0 µg  kg^−1^) and ^3^H-D-S-CPP = 16.2 × 10^7^ dpm  kg^−1^ (3.5 µg  kg^−1^) and ^3^H-IgGs = 1.3 × 10^7^ dpm  kg^−1^ (18.5–19.9 µg  kg^−1^) ± non-radiolabeled L-S-CPP (1.4 mg  kg^−1^). Injected doses were selected to provide significant detection in counting disintegrations per minute (dpm) well above background measures.

Observation of the plasma-level time curve indicated that CPPs declined biexponentially following a two-compartment model kinetic with 2 phases: distribution (fast initial decline in blood concentrations) and elimination (slower subsequent decline in blood concentrations) (Fig. 1). By contrast, IgGs plasma-level time curve was linear following an i.v. injection, where the i.v. bolus entered the bloodstream directly and declined linearly as a single first-rate process (Fig. 2).

In the biexponential model used for S-CPPs, C_0_ was extrapolated after a logarithm transformation of the distribution data while, for IgG, C_0_ was extrapolated from the linear curve at the y-axis intercept. Of note, after an i.v. injection, C_0_ is equivalent to C_max_. The other PK parameters such as the apparent distribution and elimination half-life (*T*_1/2_α,*T*_1/2_β), plasma clearance (Cl), central volume of distribution (V_C_ = D_0_/C_0_), and the area under the concentration *versus* time curve (AUC)^62,63^ were generated using Excel add-in PK Solver^64^.

Linear kinetic of IgG obtained from the plasma-level time curve after i.v. bolus, is best described by Eq. (1) mathematical expression, where *ke* represents the distribution rate constant dependent on the amount or concentration of IgG present, and C_0_ the initial IgG concentration.

1 $$C\left(t\right)={C}_{0}.{e}^{-ke.t}$$

Biexponential kinetic of S-CPPs followed a two-compartment model corresponding to Eq. 2, where *C*(*t*) is the plasma concentration at time *t*; *A* and *B* are intercept terms (distribution and elimination coefficients which exhibit two compartments); α is the distribution rate constant; and *β* is the elimination rate constant^65,66^.

2 $$C\left(t\right)=A.{e}^{-\alpha t}+B.{e}^{-\beta t}$$

### Biodistribution

Mice (10-week-old males weighing ≈40 g) were injected in the caudal vein at T_0_ and sacrificed by cold-PBS intracardiac perfusion 1 or 5 h post injection under deep anesthesia with ketamine/xylazine intraperitoneal (i.p.) injection (300 mg  kg^−1^ ketamine, 30 mg  kg^−1^ xylazine). Mice were used because our Animal Ethics Committee encourages the use of the smallest animal species possible and there was no methodological advantage in using rats. The following preparations were compared: tritiated S-CPP in its 2 enantiomeric forms, ^3^H-L-S-CPP and ^3^H-D-S-CPP, and ^3^H-IgG_antiNUP,_ alone or in combination with unlabeled L-S-CPP, D-S-CPP, or scrambled peptide (SCR). Doses injected were: ^3^H-L-S-CPP = 10.4 µg  kg^−1^, ^3^H-D-S-CPP = 15.9 µg  kg^−1^, ^3^H-IgG = 82.5 µg  kg^−1^ and mean dose of unlabeled L/D-S-CPP or scrambled peptide ≈ 3.6 mg  kg^−1^. Estimated C_0_ in circulation are shown in Table 2. The scrambled peptide had 30 amino acids, like S-CPPs, but without any CPP property.

Samples from organs (brain, lung, heart, liver, spleen, pancreas, gastrocnemius muscle and kidney) with an approximate weight of 150 mg were collected, weighed and solubilized with SOLVABLE at 50 °C overnight for quantification of tritiated molecules. To prevent quenching, most organs (lung, heart, kidney, spleen and liver) were incubated with 30% hydrogen peroxide (H_2_O_2_) (200 µL) after solubilization. Blood samples were centrifuged, and both plasma and organs were counted after addition of 9 ml of Ultima Gold scintillation cocktail. Apparent distribution volumes (V_D_) in µl  g^−1^ were computed by dividing the concentration in organs relative to weight (dpm  g^−1^) by the concentration in the blood relative to volume (dpm  µl^−1^). The use of V_D_ allowed data normalization with plasma concentration for each animal. TCA-precipitated radioactivity ranged between 90 and 100% in blood samples after 1 h.

### Toxicology

Mice (10-week-old male CD-1) were injected *bis in die* (BID) during 5 days, in the morning and the afternoon with a gap of 6 h between the two injections, alternately into the tail vein and the retro-orbital region (Table 1). The injected dose was 3.6 mg  kg^−1^ for each form of S-CPP. Animals were sacrificed by cold-PBS intracardiac perfusion. Blood hematology and biochemical parameters were determined at our hematology facilities (Heska Element/Dri-Chem). To assess pro- and anti-inflammatory cytokines, Bioplex kits for 8 cytokines were used (Biorad Mouse Cytokine 8-plex Assay #M60000007A).

### In situ cerebral perfusion to assess the passage through the blood–brain barrier (BBB).

The in situ cerebral perfusion (ISCP) technique allows to measure the volume of distribution in the brain and transport coefficient (Clup) across the BBB of compounds after an intracarotid perfusion. Since 100% of the perfusate reaches the BBB, distribution and transport parameters can be readily determined. ISCP was conducted as previously described^67–70^. Briefly, 4-month-old male balb/c mice were anesthetized by intraperitoneal injection of xylazine/ketamine (8/140 mg  kg^−1^). The right common carotid artery was catheterized to perfuse 0.2–0.8 µCi  ml^−1^ of ^3^H-L-S-CPP (corresponding to doses of 0.2, 0.4 and 0.8 µg/mouse and concentrations of 0.08, 0.16 and 0.32 µg/ml) at 2.5 ml min^−1^ in a bicarbonate buffered physiological saline, co-perfused with ^14^C-sucrose (0.12 µCi  ml^−1^) to quantify the vascular space and to assess the physical integrity of the BBB. The measured vascular space remained under 20 µl  g^−1^ in the present work confirming physical integrity of the BBB. The perfusion was terminated by decapitating the mouse at selected time (60 s). The right cerebral hemispheres and aliquots of the perfusion fluid were collected and weighed. Tissue samples were digested in 1 mL of Solvable at 50 °C overnight, and then cooled to room temperature and mixed with 9 mL of Ultima Gold scintillation cocktail. Both isotopes were counted in a Hidex 300 SL Liquid Scintillation Counter. The brain transport coefficient (Clup, μL  g^−1^  s^−1^) of ^3^H-L-S-CPP was calculated from the measured volume of distribution (V_brain_, µl  g^−1^) of ^3^H-L-S-CPP, corrected with the vascular space (V_vasc_, μl  g^−1^) determined with ^14^C-sucrose. The following equation was used:

$${\text{Clup}}\;\left( {{\mu l}\;{\text{g}}^{{ - {1}}} \;{\text{s}}^{{ - {1}}} } \right){ = }\frac{{{\text{Vvrain}}}}{{\text{T}}}{ , }\quad {\text{where}} {\text{V}}_{{{\text{brain}}}} = \, \left( {{\text{X}}_{{{\text{L}} - {\text{S}} - {\text{CPP}}}} /{\text{C}}_{{{\text{L}} - {\text{S}} - {\text{CPP}}}} } \right) - \, \left( {{\text{X}}_{{{\text{sucrose}}}} /{\text{C}}_{{{\text{sucrose}}}} } \right).$$

V_brain_ (µl  g^−1^) represents the apparent volume of distribution of study compound, T (s) is the time, X (dpm  g^−1^) is the quantity of radioactivity found in the brain corresponding to the injected molecule and C is the concentration (dpm  µl^−1^) in the perfusion fluid.

In addition, extravascular % of ^3^H-L-S-CPP corresponding to the fraction not remaining in the vascular compartment was estimated following this equation:

$${\text{Extravascular }}\left( {\text{\%}} \right){ = }\frac{{\text{X}}_{{{\text{L}} - {{\text{S}}} - {{\text{CPP}}} - {{\text{( Vvasc - CL - S - CPP)}}}}}}{{\text{X}}_{{{\text{L}} - {{\text{S}}} - {{\text{CPP}}}}}}{ }$$

### Statistical analysis

Data are shown as mean ± standard error of the mean (SEM). When normality was verified, unpaired Student’s t-tests were used to identify significant differences between two groups. Otherwise, non-parametric Mann–Whitney tests were performed. Statistical differences between three groups or more were determined using one-way analysis of variance (ANOVA) followed by Tukey’s multiple comparisons tests or, when variances were not equivalent according to a Bartlett’s test, Welch-ANOVA followed by Dunnett’s post-hoc tests. Statistical analysis of biodistribution data were performed after logarithmic transformation. All of the tests were two-tailed, and statistical significance was set as follows: *P < 0.05; **P < 0.01; ***P < 0.001.

## Data Availability

The datasets generated during this study are available from the corresponding author on reasonable request.
